# Mechanistic insights into Guizhi Fuzi decoction for lumbar disc herniation: Integrating network pharmacology and bioinformatics approach

**DOI:** 10.1097/MD.0000000000041917

**Published:** 2025-03-21

**Authors:** Jiafeng Peng, Hongxing Zhang, Huaize Wang, Qianqian Meng, Danyang Li, Minglei Gao, Yingchun Li, Xingfu Ma, Li Xia, Ran Xu, Junchen Zhu

**Affiliations:** a The Second Affiliated Hospital of Anhui University of Chinese Medicine, Hefei, Anhui, China; b Graduate School of Anhui University of Traditional Chinese Medicine, Hefei, Anhui, China.

**Keywords:** Guizhi Fuzi decoction, herbal medicine, lumbar disc herniation, network pharmacology

## Abstract

The ancient Chinese medical texts have recorded Guizhi Fuzi decoction (GZFZT) as a therapeutic intervention for lumbar disc herniation (LDH), and its clinical efficacy has been validated in medical practice. This research endeavor is specifically designed to delve into and elucidate its precise underlying mechanisms of action, leveraging the sophisticated methodologies of network pharmacology and bioinformatics. In this study, we used the Traditional Chinese Medicine Systems Pharmacology Database and Analysis Platform to extract active compounds and targets from the traditional Chinese medicine GZFZT. Subsequently, we integrated LDH disease target information from DisGeNET, GeneCards, OMIM, and GEO database. By combining this with drug-effective targets, we screened for common targets. Based on these, we conducted protein–protein interaction network analysis and performed gene ontology and Kyoto Encyclopedia of Genes and Genomes pathway enrichment analyses on core targets to explore LDH treatment pathways. Finally, we used molecular docking to evaluate potential targets and compounds, identifying the optimal core protein-compound complex. Our study identified 154 active compounds and 230 corresponding targets of GZFZT. Additionally, we collected a total of 1492 LDH disease targets. Topological analysis of the protein–protein interaction network for common drug-disease targets revealed 6 core targets: TNF, STAT3, MAPK1, IL6, MAPK3, and AKT1. Gene ontology enrichment analysis indicated that the mechanism of action of GZFZT is associated with inflammatory responses, apoptotic processes, and oxidative stress states. Kyoto Encyclopedia of Genes and Genomes enrichment analysis suggested that the mechanism of action of GZFZT is closely related to genes involved in the AGE-RAGE and IL-17 signaling pathways. Molecular docking results demonstrated that the selected compounds exhibit strong binding affinity to the targets, indicating their good biological activity. This study unveils novel insights into the active ingredients, targets, and signaling pathways of Guizhi Fuzi decoction in the treatment of lumbar disc herniation. Furthermore, this study suggests that the 3 bioactive components of Guizhi Fuzi decoction (naringenin, β-sitosterol, and stigmasterol) may exert their therapeutic effects on lumbar disc herniation by specifically targeting MAPK3.

## 1. Introduction

**Figure 1.**
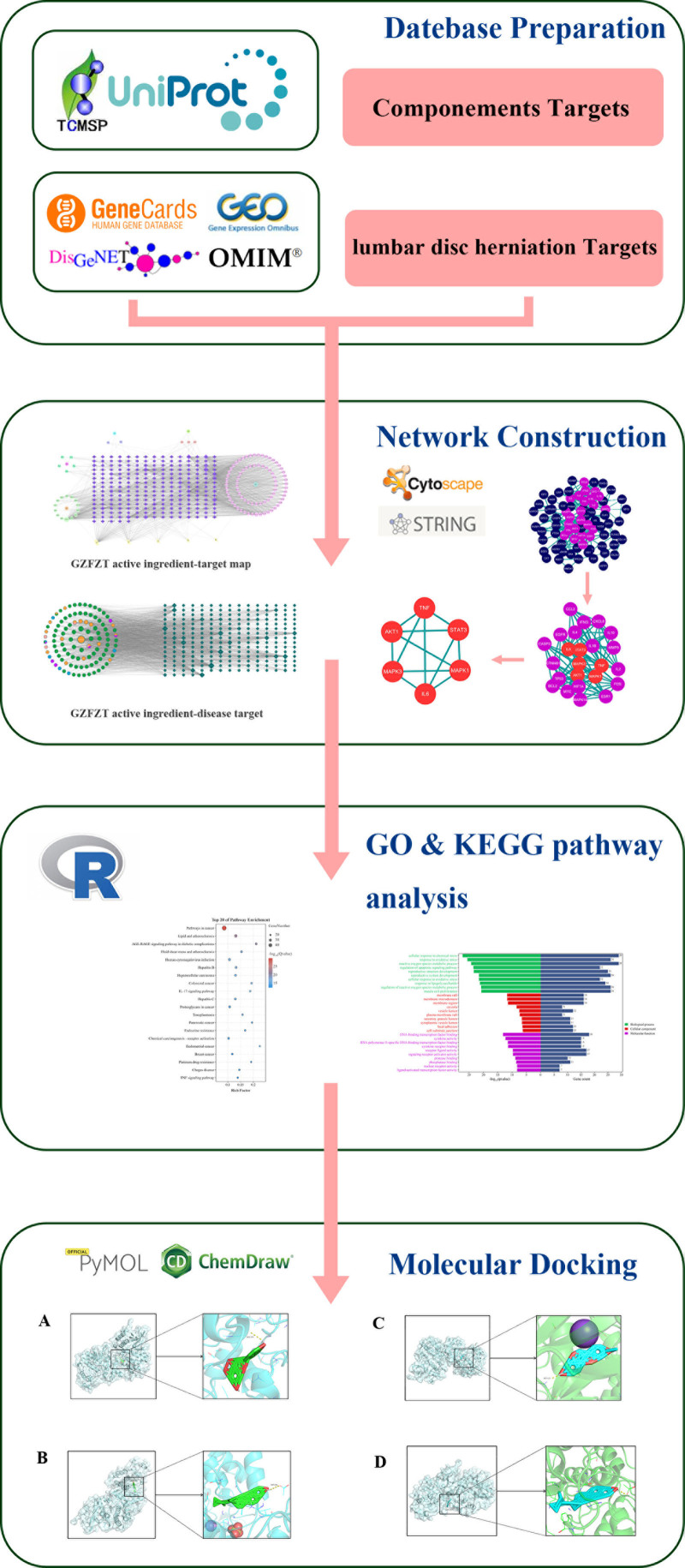
The network pharmacology of GZFZT is divided into four parts: molecular docking validation, network construction, GO and KEGG pathway analysis, and database preparation. GO = gene ontology, GZFZT = Guizhi Fuzi decoction, KEGG = Kyoto Encyclopedia of Genes and Genomes.

Lumbar disc herniation (LDH) refers to the displacement of the nucleus pulposus (NP) through the annulus fibrosus beyond the normal intervertebral disc space, resulting in irritation or compression of adjacent spinal nerve roots.^[1]^ Clinically, patients may present with lumbar pain, which can be accompanied by radiating pain and sensory decrement in the lower limbs. In severe cases, they may develop cauda equina syndrome or become unable to walk.^[2]^ The treatment of LDH can be categorized into nonsurgical and surgical approaches. Nonsurgical treatments, including bed rest, exercise, physiotherapy (traction, acupuncture, massage, etc), and pharmacological therapy (nonsteroidal anti-inflammatory drugs, muscle relaxants, etc).^[3]^ It is noteworthy that most patients experience spontaneous regression of the herniated disc tissue, and approximately 60% to 90% of LDH cases can be managed with conservative treatment strategies.^[4,5]^ Therefore, in-depth research on conservative treatment methods is essential. In TCM theory, LDH is referred to as “Bi syndrome,” typically caused by Qi stagnation, blood stasis, dampness-cold, or deficiency in liver and kidney functions, and oral administration of traditional Chinese medicine is considered one of the important ways to treat LDH.^[6]^ The traditional Chinese medicine formula Guizhi Fuzi Decoction (GZFZT), recorded in ancient literature for the treatment of “bi syndrome,” consists of “Guizhi” (Cassia Twig), “Fuzi” (Monkshood Root), “Shengjiang” (Fresh Ginger), “Dazao” (Jujube), and “Gancao” (Licorice Root). Studies have indicated that Guizhi and Fuzi can alleviate the symptoms of lumbar pain and reduce inflammation in the lumbar spine.^[7,8]^ Moreover, pharmacological research on other components of GZFZT suggests that they are also associated with the pathogenesis of LDH.^[9–11]^ In modern medicine, compared with nonsteroidal anti-inflammatory and analgesic drugs and muscle relaxants, researchers have found that GZFZT can equally improve symptoms in LDH patients without adverse reactions, and its long-term effects are more significant and stable.^[12]^ Furthermore, for LDH patients undergoing surgical treatment, GZFZT can facilitate their postoperative recovery and enhance the therapeutic efficacy.^[13]^ Importantly, due to the complexity of patients’ conditions and the diversity of disease mechanisms, a single drug is often insufficient to address the diverse manifestations of LDH.^[14]^ However, GZFZT utilizes the 4 properties (cold, hot, warm, and cool) and 5 tastes (sweet, sour, bitter, pungent, and salty) of 5 herbal medicines to provide targeted symptomatic treatment for the complex conditions of LDH patients.^[15]^ Currently, despite the clinical validation of the overall therapeutic efficacy of GZFZT, there is a lack of in-depth research into the mechanism by which the individual herbal components of GZFZT synergistically treat LDH. Therefore, our team has employed a comprehensive approach using network pharmacology, bioinformatics, and molecular docking techniques to explore the active ingredients and target proteins of GZFZT, and to elaborate on its mechanism of action in treating LDH. We hope that our research can provide a foundation for future studies on LDH treatment, offer more therapeutic insights to clinicians, and ultimately benefit patients. The research process is illustrated in Fig 1.

## 2. Methods

### 2.1. Identification of potential therapeutic targets of GZFZT

Traditional Chinese Medicine Systems Pharmacology Database and Analysis Platform (TCMSP, https://tcmsp-e.com/) is a database of systems pharmacology for Traditional Chinese Medicine, used to screen the active components and potential therapeutic targets of GZFZT. This database offers valuable connections between herbal treatments, disease targets, and diseases, making it an essential resource for TCM research.^[16]^ The screening criteria for GZFZT herbs included an oral bioavailability ≥ 30% and drug-likeness ≥ 0.18%. These parameters are essential for evaluating the ADME characteristics and are used to screen active compounds for drug selection. The effective active components and protein targets of the 5 herbs in GZFZT were retrieved using the keywords “Guizhi,” “Fuzi,” “Shengjiang,” “Dazao,” and “Gancao.” The targets were standardized using the UniProt protein database (https://www.uniprot.org).

### 2.2. GEO differential gene expression analysis

Search the GEO database (www.ncbi.nlm.nih.gov/geo/) using the keyword “lumbar disc herniation.” Select an appropriate GEO data chip and download the corresponding GSE files for subsequent analysis using R. Identify differentially expressed genes using the Limma package with criteria of logFC > 1 and *P* < .05. Use the ggplot2 package to create a volcano plot of the differentially expressed genes and the pheatmap package to generate heatmaps for the top 10 upregulated and downregulated differentially expressed genes.

### 2.3. Collecting therapeutic targets for LDH

The targets associated with LDH were obtained by searching the DisGeNET, GeneCards, and OMIM databases using the keyword “lumbar disc herniation.” These targets were integrated with the differentially expressed genes from GEO. The UniProt database was used to convert target names to gene symbols and eliminate duplicates.

### 2.4. Active ingredient-disease target network construction of Chinese medicine

The potential targets of active ingredients in GZFZT intersect with potential targets for LDH. Networks and type files were constructed and imported into Cytoscape 3.8.0 to build the GZFZT active ingredient-disease target network diagram.

### 2.5. Analysis of PPI network, GO and KEGG

The intersecting target genes were carefully selected and imported into the STRING database with a minimum interaction score > 0.9, specifying the species as ``Homo sapiens’’. Using Cytoscape 3.8.0 software, a protein–protein interaction (PPI) network was constructed, taking into account hidden nodes. Gene ontology (GO) function and Kyoto Encyclopedia of Genes and Genomes (KEGG) pathway analyses were conducted through online bioinformatics platforms (https://www.bioinformatics.com.cn/, https://www.omicshare.com/) to explore biological data of core target genes. This analysis provides insights into the complex regulatory mechanisms and interactions among these genes.

### 2.6. Molecular docking

Molecular docking is a critical technique in network pharmacology used to validate compound-target interactions and determine binding affinity. Based on the degree values in the PPI network, 6 potential target proteins were selected for molecular docking studies. The PDB files of the main targets were obtained from the RCSB database (https://www.rcsb.org), while the 3D structures of the primary active compounds in GZFZT were sourced from the PubChem database. Pymol software was used to prepare the core target proteins by removing solvent molecules, and AutoDock Tools 1.5.7 was employed for further hydrogenation and charging. The core target proteins and active compounds were saved as “pdbqt” format files, and AutoDock Vina was utilized to set the position and size of the docking box for the docking process.

## 3. Results

### 3.1. Potential therapeutic targets for GZFZT

**Figure 2.**
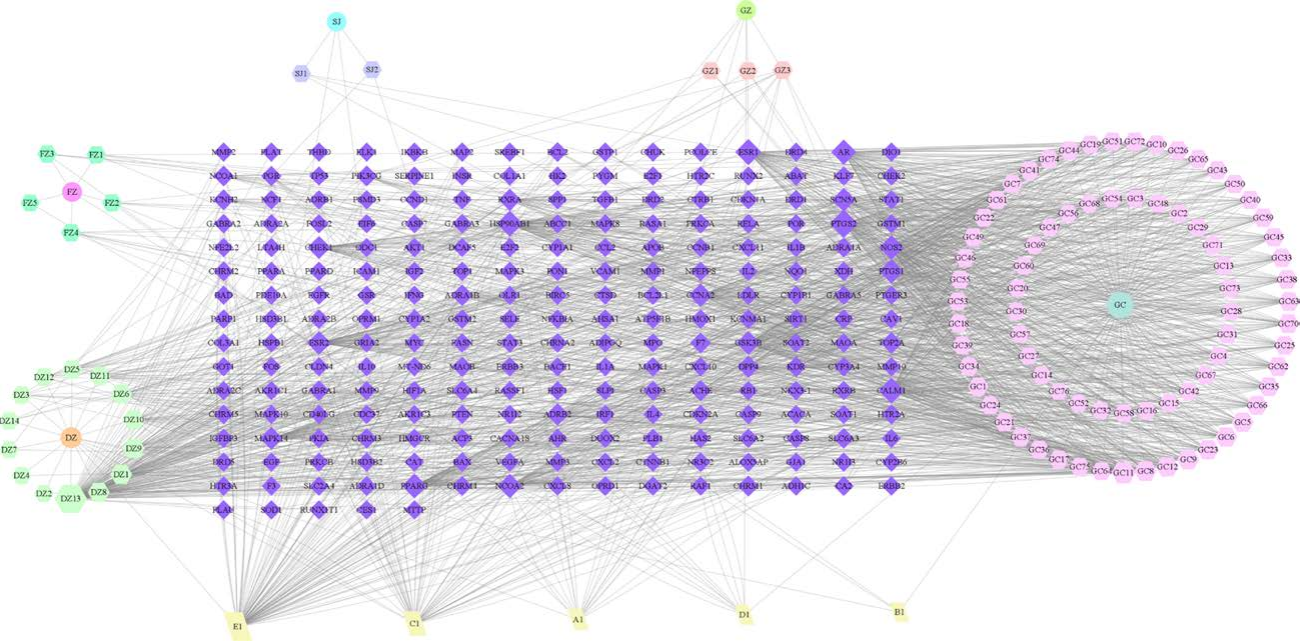
GZFZT active ingredient-target map. GZFZT = Guizhi Fuzi decoction.

Using the TCMSP database, 154 active compounds were screened, including 7 from Guizhi, 21 from Fuzi, 29 from Dazao, 5 from Shengjiang, and 92 from Gancao. Detailed data can be found in Table S1, Supplemental Digital Content, http://links.lww.com/MD/O583. This analysis identified 230 drug targets. The data were visualized using Cytoscape 3.8.0, generating a network diagram of the “GZFZT Active Ingredients-Targets” relationship (Fig. 2). Different shapes and colors represent herbal components and target genes respectively. Diamonds indicate drug targets, and circles represent active ingredients. Guizhi, Fuzi, Shengjiang, Dazao, and Gancao are represented in green, purple, blue, orange, and cyan, respectively.

### 3.2. GEO differential gene analysis

**Figure 3.**
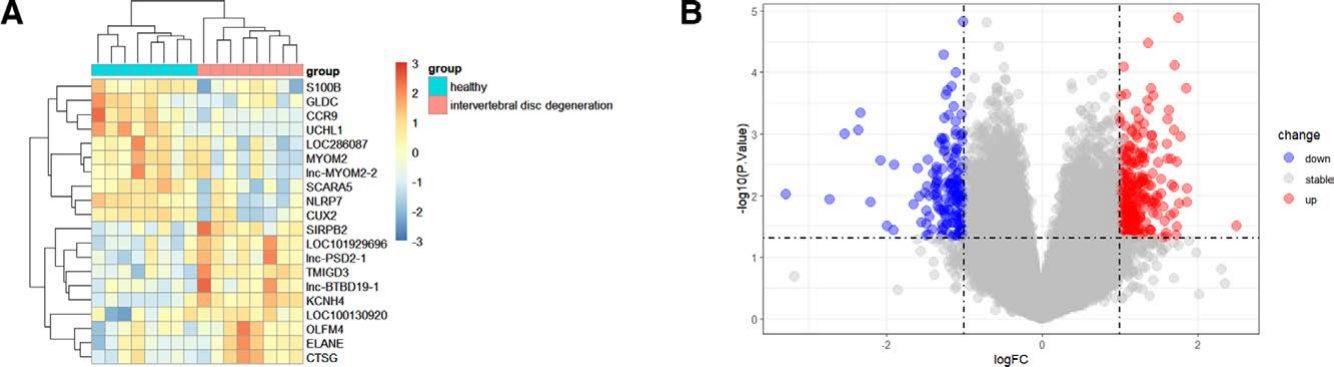
(A) Heatmap of the top 10 upregulated and downregulated differentially expressed genes related to LDH in the GEO dataset (GSE124272). (B) Volcano plot of genes related to LDH in the GEO dataset (GSE124272). LDH = lumbar disc herniation.

Download the gene expression data for LDH from the GEO database (GSE124272), which includes 8 samples from normal individuals and 8 samples from LDH patients. A total of 417 differentially expressed genes were identified, of which 251 were upregulated and 166 were downregulated, as shown in Fig. 3B. Detailed data can be found in Table S2, Supplemental Digital Content, http://links.lww.com/MD/O584. The heatmap of the top 10 upregulated and downregulated differentially expressed genes is shown in Fig. 3A, with information on these genes provided in Table 1.

**Table 1:** GEO dataset analysis of differential gene information.

Gene symbol	Protein name	LogFC	*P* value	Change
OLFM4	Olfactomedin 4	2.496978592	.030965851	Up
ELANE	Elastase, neutrophil expressed	1.860879822	.012819914	Up
lnc-BTBD19-1	lnc-BTBD19-1:1	1.86069558	.007687096	Up
TMIGD3	Transmembraneand immunoglobulin domain containing 3	1.858415865	.000184808	Up
LOC101929696	Uncharacterized LOC101929696	1.782427606	.001120253	Up
lnc-PSD2-1	lnc-PSD2-1:1	1.766702631	.013625379	Up
KCNH4	Potassium channel, voltage gated eag related subfamily H, member 4	1.752769705	.0000134	Up
SIRPB2	Signal-regulatory protein beta 2	1.735028951	.002836571	Up
CTSG	Cathepsin G	1.733010388	.032621188	Up
LOC100130920	Uncharacterized LOC100130920	1.730522881	.044798355	Up
CUX2	Cut-like homeobox 2	1.893035268	.003200032	Down
GLDC	Glycine dehydrogenase (decarboxylating)	1.910235826	.036778545	Down
lnc-MYOM2-2	lnc-MYOM2-2:1	1.991918312	.031667697	Down
SCARA5	Scavenger receptor class A, member 5	2.069630316	.002740355	Down
S100B	S100 calcium binding protein B	-2.20282593	.013001029	Down
UCHL1	Ubiquitin carboxyl-terminal esterase L1 (ubiquitin thiolesterase)	2.331049551	.00045836	Down
CCR9	Chemokine (C-C motif) receptor 9	2.360747562	.000884111	Down
NLRP7	NLR family, pyrin domain containing 7	2.531252895	.001014719	Down
LOC286087	Uncharacterized LOC286087	2.723102554	.011643299	Down
MYOM2	Myomesin 2	3.290562835	.009700781	Down

### 3.3. Collecting therapeutic targets for LDH

**Figure 4.**
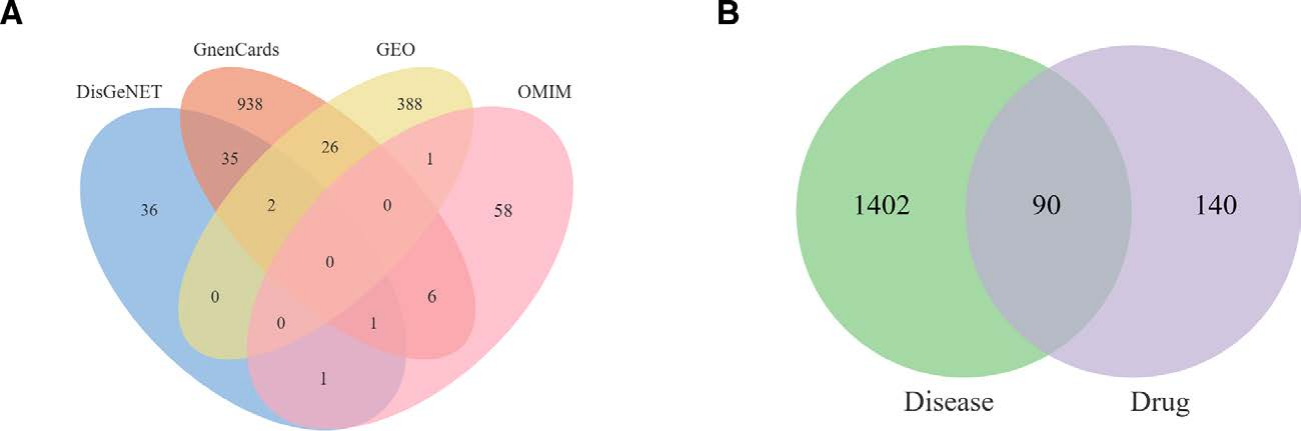
(A) Venn diagram of LDH targets from three disease databases and the GEO dataset. (B) Venn diagram comparing targets from disease databases with therapeutic targets of GZFZT. GZFZT = Guizhi Fuzi decoction, LDH = lumbar disc herniation.

Integrating the GEO database with Disgenet, Genecards, and OMIM databases, 1492 LDH targets were identified, as shown in Fig. 4A. Detailed information is available in Table S3, Supplemental Digital Content, http://links.lww.com/MD/O585. After removing duplicate targets, the intersection of LDH active compound targets and disease targets resulted in the identification of 90 common targets. These 90 targets are considered the primary therapeutic targets for LDH, as shown in Fig. 4B. Detailed information is available in Table S4, Supplemental Digital Content, http://links.lww.com/MD/O586.

### 3.4. Active ingredient-disease target network construction of Chinese medicine

**Figure 5.**
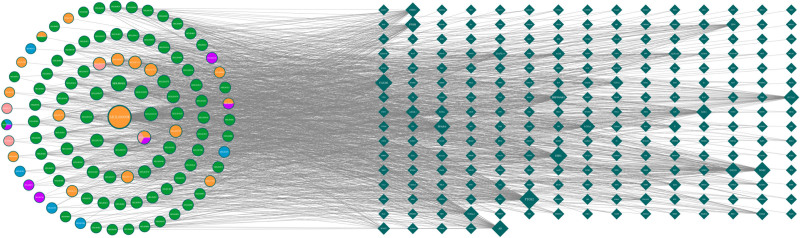
GZFZT active ingredient-disease target. GZFZT = Guizhi Fuzi decoction

Using Cytoscape 3.8.0 software, we constructed a network of therapeutic targets for GZFZT active components, comprising 239 nodes and 984 edges. The network was subjected to visual analysis, with results shown in Fig 5. In the network diagram, squares represent disease targets, while circles represent medicinal herb components. Gui Zhi, Fu Zi, Sheng Jiang, Da Zao, and Gan Cao are represented in purple, blue, pink, orange, and green, respectively. The main active components identified for GZFZT include quercetin, kaempferol, 7-methoxy-2-methyl isoflavone, naringenin, formononetin, licochalcone A, isorhamnetin, medicarpin and stigmasterol, as shown in Table 2.

**Table 2:**
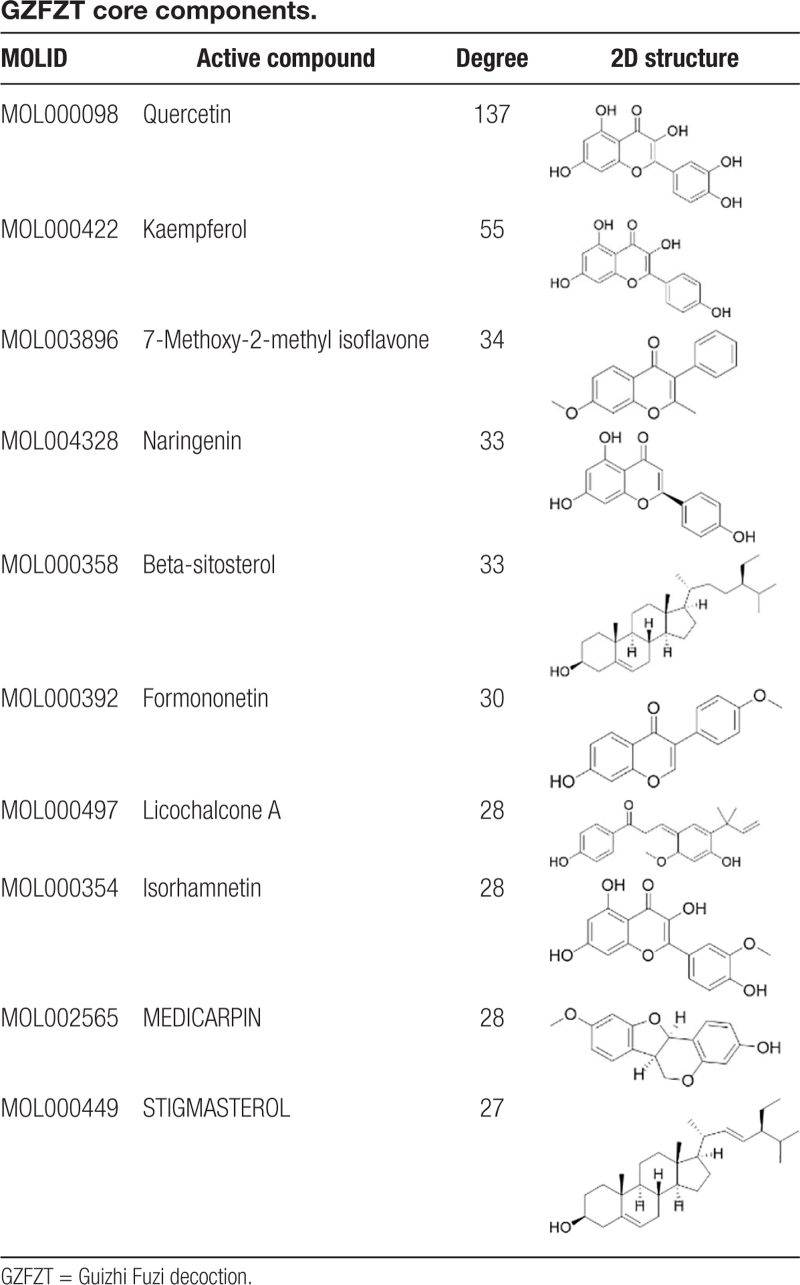
GZFZT core components.

### 3.5. Analysis of PPI network, GO and KEGG

**Figure 6.**
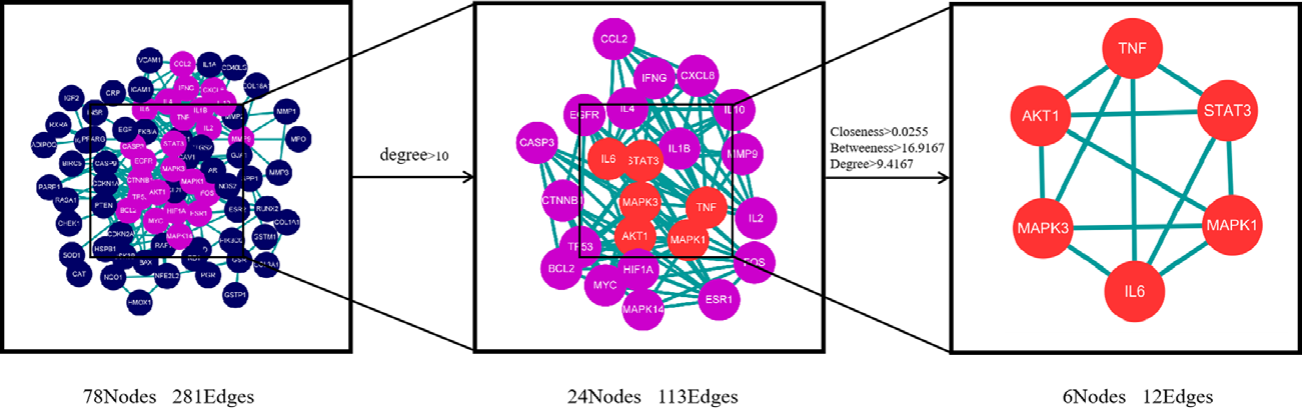
PPI core network of GZFZT for LDH. GZFZT = Guizhi Fuzi decoction, LDH = lumbar disc herniation, PPI = protein–protein interaction.

**Figure 7.**
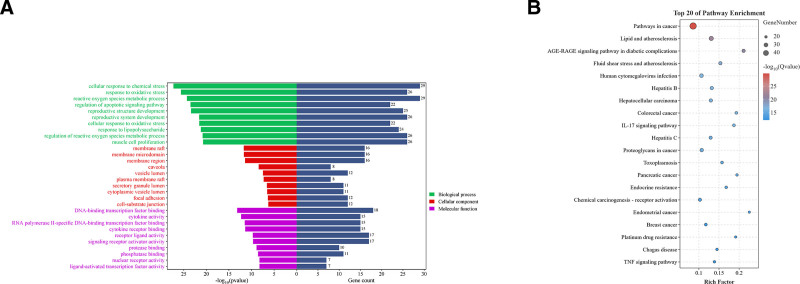
(A) Results of GO enrichment analysis. (B) Results of KEGG enrichment analysis.

To obtain the PPI network, we analyzed 90 common targets using the String database (https://cn.string-db.org/), resulting in a network with 78 nodes and 281 edges as shown in Fig. 6. This network reveals relationships between proteins. We conducted topological analysis of potential targets using the Cyto-CAN plugin and selected the top 6 targets based on their degree values, as shown in Table 3. Using R language, we performed GO and KEGG enrichment analyses on these targets, revealing their involvement in various biological processes, cellular components, and molecular functions, indicating their therapeutic potential, as illustrated in Fig. 7A. KEGG enrichment analysis emphasized their significance in multiple signaling pathways, suggesting their potential for tailored treatments against different diseases, as depicted in Fig. 7B.

**Table 3:** Detailed information of 6 targets.

No	Uniprot ID	Gene symbol	Protein name	Degree
1	P05231	IL6	Interleukin-6	13
2	P27361	MAPK3	Mitogen-activated protein kinase 3	12
3	P40763	STAT3	Signal transducer and activator of transcription 3	15
4	P28482	MAPK1	Mitogen-activated protein kinase 1	12
5	P01375	TNF	Tumor necrosis factor	13
6	P31749	AKT1	RAC-alpha serine/threonine-protein kinase	12

### 3.6. Molecular docking verification

**Figure 8.**
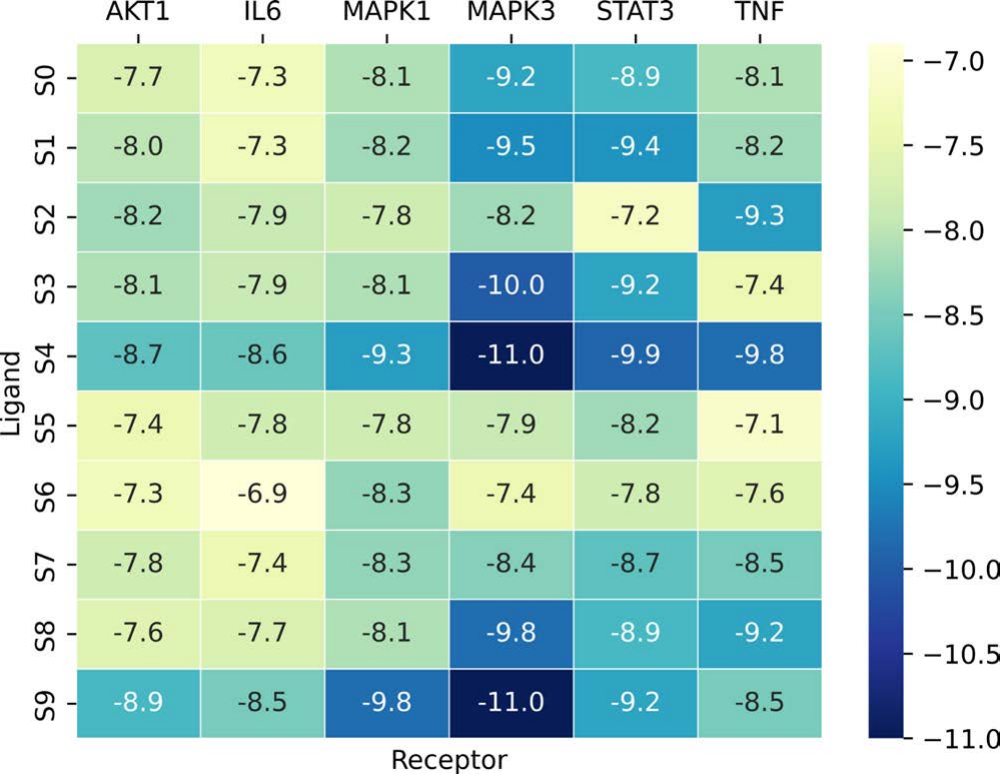
Heat map of the molecule docking grade. Chinese medicine’s key objectives and active compounds’ binding energy (kcal/mol).

**Figure 9.**
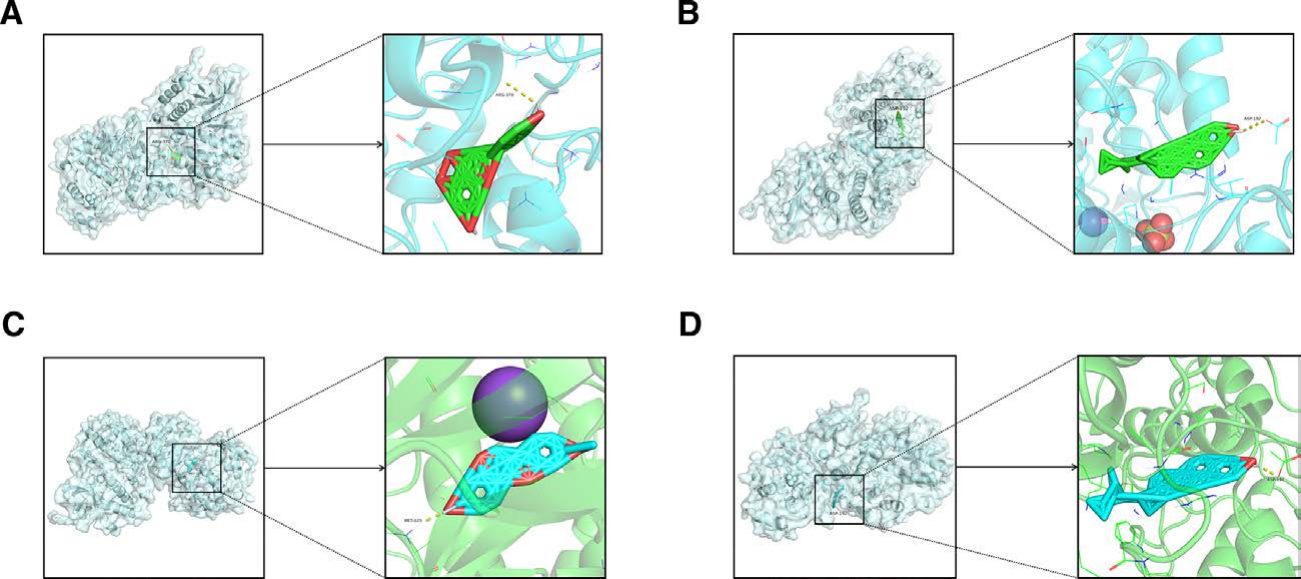
Docking results of main targets with specific active compounds. (A) Naringenin-MAPK3. (B) β-Sitosterol-MAPK3B. (C) Medioresinol-MAPK3. (D) Stigmasterol-MAPK3.

Molecular docking was employed to verify interactions between screened active compounds and targets in the context of LDH using bioinformatics. High-ranking crucial targets including IL6, MAPK3, STAT3, MAPK1, TNF, and AKT1 were selected from the PPI network results for docking analysis with 10 compounds identified from the GZFZT active ingredient-disease target network. Two-dimensional structures of the main active ingredients were obtained from the TCMSP and PubChem databases, followed by energy minimization and preparation of small molecule ligands using Chem3D software. Protein receptors were prepared by removing water molecules and ligands using PYMOL software. The binding energies (kcal/mol) between the main targets and active compounds were determined, demonstrating their binding affinities as shown in Fig. 8 and Table 4. Finally, molecular docking of ligands with receptors was conducted using AutoDock software. Compounds such as naringenin, β-sitosterol, medioresinol, and stigmasterol exhibited strong binding with core targets, while the MAPK3 active compound showed good binding with disease targets. Figure 9 displays 4 functional groups with favorable docking results, indicating a higher likelihood of molecular interactions. These results attribute enhanced stability of binding molecules due to lower binding energies between small molecule ligands and protein receptors.

**Table 4:** Binding degree of MAPK3 with corresponding compounds.

No	Compounds Mol ID	Compounds name	Target	Affinity (kcal/mol)
S0	MOL000098	Quercetin	MAPK3	‐9.2
S1	MOL000422	Kaempferol	MAPK3	‐9.5
S2	MOL003896	7-Methoxy-2-methyl isoflavone	MAPK3	‐8.2
S3	MOL004328	Naringenin	MAPK3	‐10.0
S4	MOL000358	Beta-sitosterol	MAPK3	‐11.0
S5	MOL000392	Formononetin	MAPK3	‐7.9
S6	MOL000497	Licochalcone a	MAPK3	‐7.4
S7	MOL000354	Isorhamnetin	MAPK3	‐8.4
S8	MOL002565	Medicarpin	MAPK3	‐9.8
S9	MOL000449	Stigmasterol	MAPK3	‐11.0

## 4. Discussion

In clinical practice, several traditional Chinese medicine formulas, such as Bu Yang Huan Wu Tang and Bu Shen Huo Xue Fang, have been effectively used for the treatment of LDH.^[17,18]^ GZFZT, rooted in ancient medical literature, has demonstrated efficacy in the treatment of LDH, akin to traditional Chinese medicine prescriptions previously mentioned. According to the theory of traditional Chinese medicine, the combination of Fuzi with Guizhi serves to warm yang and disperse cold, while the synergy of Shengjiang, Dazao, and Gancao harmonizes qi and blood, enhances the righteous qi, thereby alleviating patients’ symptoms and strengthening their constitution. To further explore the molecular mechanisms underlying GZFZT’s therapeutic effects on LDH, we have integrated bioinformatics, network pharmacology approaches, and molecular docking techniques, aiming to provide a scientific basis for optimizing clinical treatment strategies.

The network diagram of GZFZT active ingredient-disease targets in Fig. 5 shows a total of 105 components. Studies have shown that compounds such as quercetin, kaempferol, 7-methoxy-2-methylisoflavone, naringenin, and β-sitosterol exert effects through various mechanisms. These mechanisms include anti-inflammatory, antioxidative stress, anti-osteoporosis, neuroprotection, and promoting extracellular matrix (ECM) homeostasis.^[19,20]^ Intervertebral disc degeneration (IDD) is a major cause of disc herniation and can lead to lower back pain. Research indicates that the inflammatory factor IL-1β is a critical factor in IDD, and quercetin, a major component of GZFZT, can inhibit IL-1β-induced senescence-associated secretory phenotype in NP cells while promoting ECM homeostasis, thereby improving IDD progression.^[20,21]^ Additionally, long-term use of senolytic drugs Dasatinib and Quercetin can improve disc degeneration, reduce systemic inflammation, and ameliorate adverse physical conditions associated with aging.^[19]^ Kaempferol, one of the main components of GZFZT, has various beneficial effects and is used as a nutritional supplement for its anti-inflammatory, anti-osteoporosis, antiaging, and antioxidative properties.^[22,23]^ Studies have shown that kaempferol can alleviate IDD by reducing lipopolysaccharide-induced pro-inflammatory cytokines in bone marrow-derived mesenchymal stem cells, including IL-1β, iNOS, TNF-α, and IL-6, while inhibiting NF-κB activation.^[24,25]^ Kaempferol, naringenin, β-sitosterol, formononetin, medicarpin, and licochalcone A can prevent osteoporosis; formononetin, medicarpin, and licochalcone A do so by inhibiting osteoblast apoptosis, while kaempferol, naringenin, and β-sitosterol additionally promote osteoblast formation, providing bone protection.^[26–30]^ Most studies suggest a connection between osteoporosis and disc degeneration, but the specific mechanisms remain highly controversial and warrant further investigation.^[31]^ Moreover, naringenin exhibits anti-inflammatory and analgesic effects, making it effective for pain caused by nerve damage and inflammation.^[32,33]^ Recent research shows that naringenin has neuroprotective effects and, when used in combination with hydroxypropyl-β-cyclodextrin, can improve sciatic nerve pain in mice.^[34]^ Since the mouse model is created by directly damaging the sciatic nerve, further verification is needed to determine if naringenin can alleviate pain caused by sciatic nerve compression due to disc herniation. Stigmasterol, a plant-derived sterol, has antioxidant and anti-inflammatory properties and is effective in treating osteoarthritis. In vivo studies indicate that stigmasterol can upregulate the expression of anti-inflammatory cytokine IL-10 through the NF-κB and MAPK pathways, improving osteoarthritis.^[35]^ Local inflammation is a significant factor in disc degeneration and herniation, thus stigmasterol has great potential for treating LDH. In summary, the main active components of GZFZT exhibit multiple mechanisms of action related to LDH. Additionally, other active ingredients, such as 7-methoxy-2-methylisoflavone and isorhamnetin, may have potential therapeutic effects in treating LDH. Exploring these components can enhance future research on LDH treatment, potentially bringing relief to patients with LDH.

Among the top 10 upregulated and downregulated differentially expressed genes, we identified a connection between SIRPB2 and LDH. SIRPB2 is a member of the SIRP receptor family. Analyzing whole blood RNA-seq transcriptome data from 25 LDH patients and healthy volunteers using WGCNA, researchers found that SIRPB2 expression was significantly upregulated and identified as one of the hub genes.^[36]^ SIRPB2 itself does not transmit activating or inhibitory signals, but when it binds to CD47 on antigen-presenting cells, it mediates cell–cell adhesion, promoting antigen-specific T cell proliferation and cytokine secretion. Therefore, SIRPB2 may play an important role in the immune response within the inflamed disc environment.^[37]^ SCARA5 is a class A scavenger receptor encoded on chromosome 8.^[38]^ Studies have found that SCARA5 expression levels in LDH patients are negatively correlated with neutrophil expression and activated mast cell expression. Researchers have speculated that the downregulation of SCARA5 in LDH patients may be related to promoting the proliferation of the NP and the occurrence of inflammation. S100B, an extracellular factor, was found to be generally upregulated in LDH patients in this study. This may be associated with S100B causing myocyte damage and inhibiting osteoblasts.^[39,40]^ OLFM4 is an antiapoptotic protein that has been reported to play a role in cell growth and inflammation, potentially promoting fibroblast-like synoviocytes proliferation or antiapoptosis, exacerbating synovial inflammation.^[41]^ OLFM4 is significantly upregulated in LDH patients, suggesting that OLFM4 could be a potential target for LDH treatment. Through bioinformatics analysis, we identified several differentially expressed genes. However, some of these genes have not been extensively studied or validated in related research. This may be due to their recent discovery, limited research, or insufficient exploration. Despite the lack of supporting literature, we believe these genes merit further investigation. They may play crucial roles in specific biological processes, disease progression, or therapeutic interventions, yet remain inadequately understood. Therefore, we recommend additional experiments and studies to gain a comprehensive understanding of the functions and significance of these under-researched genes, providing valuable insights.

PPI network analysis identified core targets, including TNF, STAT3, MAPK1, IL-6, MAPK3, and AKT1. IDD is a common degenerative disease, leading to NP collapse or herniation, causing abnormal nerve root conditions in patients.^[42]^ TNF-α is considered a key component exacerbating inflammation during disc degeneration and affecting ECM homeostasis.^[43,44]^ Studies have shown a significant increase in TNF-α expression in the intervertebral disc and adjacent muscles of discogenic low back pain group rats compared to controls.^[45]^ IL-6 is a cytokine signaling through the type I cytokine receptor complex, exhibiting both anti-inflammatory and pro-inflammatory properties.^[46]^ In addition to T cells and macrophages, IL-6 is also secreted by intervertebral disc cells.^[47]^ Research indicates that protectin DX reduces IL-6 and IL-1β mRNA levels in non-compressive lumbar disc herniation rats, promoting transforming growth factor β mRNA transcription and improving lumbar nerve root pain.^[48]^ Furthermore, IL-6 not only impacts the catabolic metabolism of NP cells but also induces TNF expression in the dorsal root ganglion and apoptosis of neuronal cells, potentially leading to abnormal pain perception.^[49–51]^ STAT is a DNA-binding protein family, and the JAK/STAT signaling pathway is activated in LDH and plays a role in inflammatory responses.^[51]^ Studies indicate that under negative regulation by SOCS3, the JAK/STAT signaling pathway can be activated by IL-6 and transmit information from the cell surface to the nucleus. Inhibiting this pathway may attenuate cell apoptosis and ECM degradation, thereby regulating the degeneration process of lumbar intervertebral disc chondrocytes.^[25,52]^ MAPK1 is considered a central regulator of various biochemical signals that modulate cellular functions such as proliferation, differentiation, and apoptosis.^[25]^ Previous research has implicated MAPK1 in the regulation of inflammation and catabolic metabolism in intervertebral disc, with MAPK1 regarded as a pivotal gene in Lumbar disc degeneration.^[53,54]^ Upregulated expression of MAPK1 inhibits NP cell proliferation, promotes NP cell senescence, ultimately leading to the development of disc degeneration and even LDH.^[55]^ Akt has 3 subtypes (AKT1, AKT2, and AKT3), all sharing a conserved structure domain with 80% sequence similarity.^[56]^ The Akt/PKB signaling pathway is considered a major mediator of cell survival, controlling cell growth, proliferation, and regulating cell movement and migration.^[57]^ Research concludes that Akt1/PKB constitutes approximately 75% of Akt/PKB total activity, and activation of only the Akt1/PKBa subtype correlates with the severity of intervertebral disc herniation.^[58–60]^ Interestingly, AKT1 and AKT3 mRNA show significant positive correlation only in intervertebral disc herniation, suggesting potential synergistic effects of these subtypes in this condition.^[60]^ In summary, GZFZT may exert its therapeutic effects on LDH by targeting relevant targets such as TNF, STAT3, MAPK1, IL-6, and AKT1.

**Figure 10.**
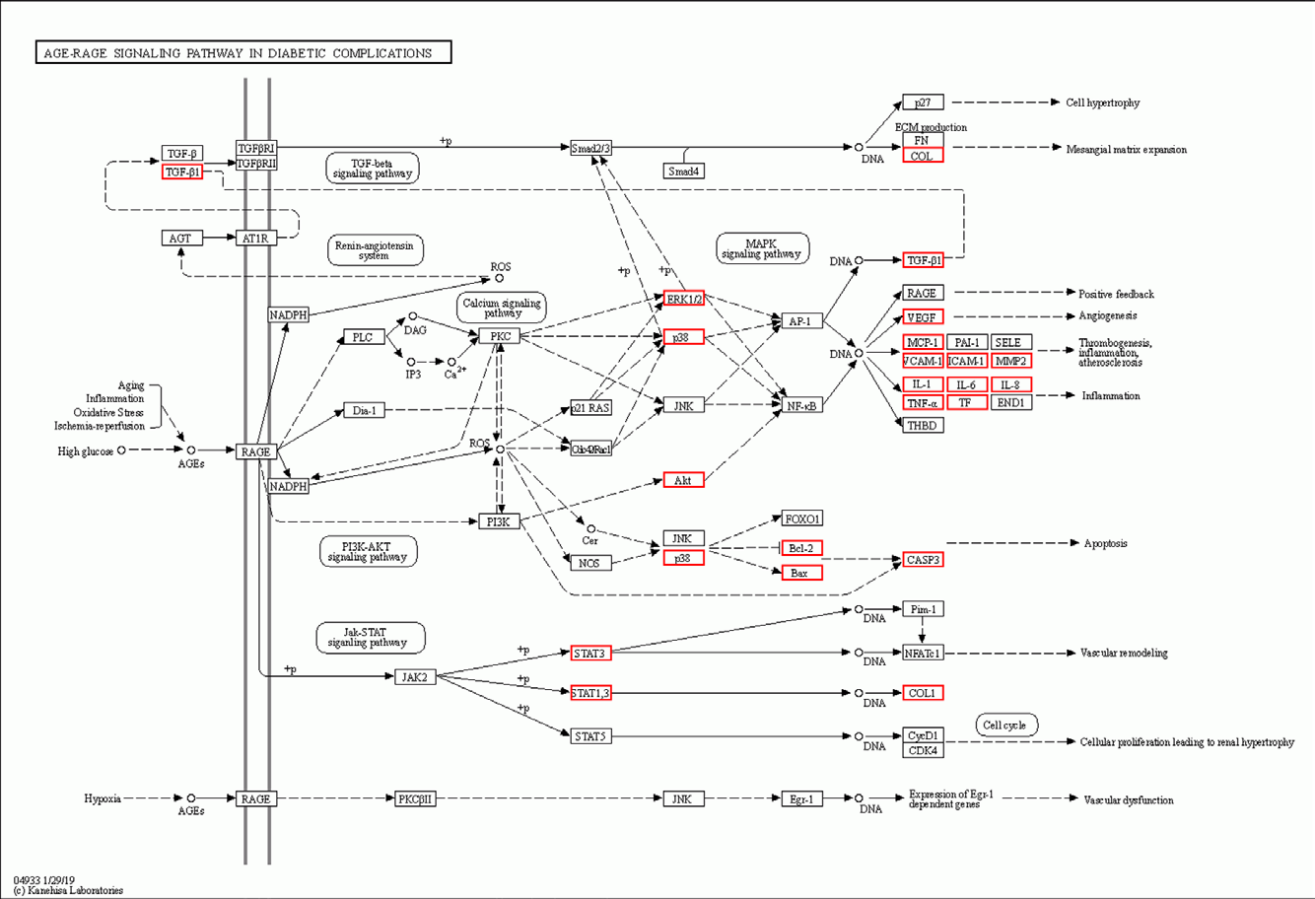
AGE-RAGE pathway (red is the core target).

**Figure 11.**
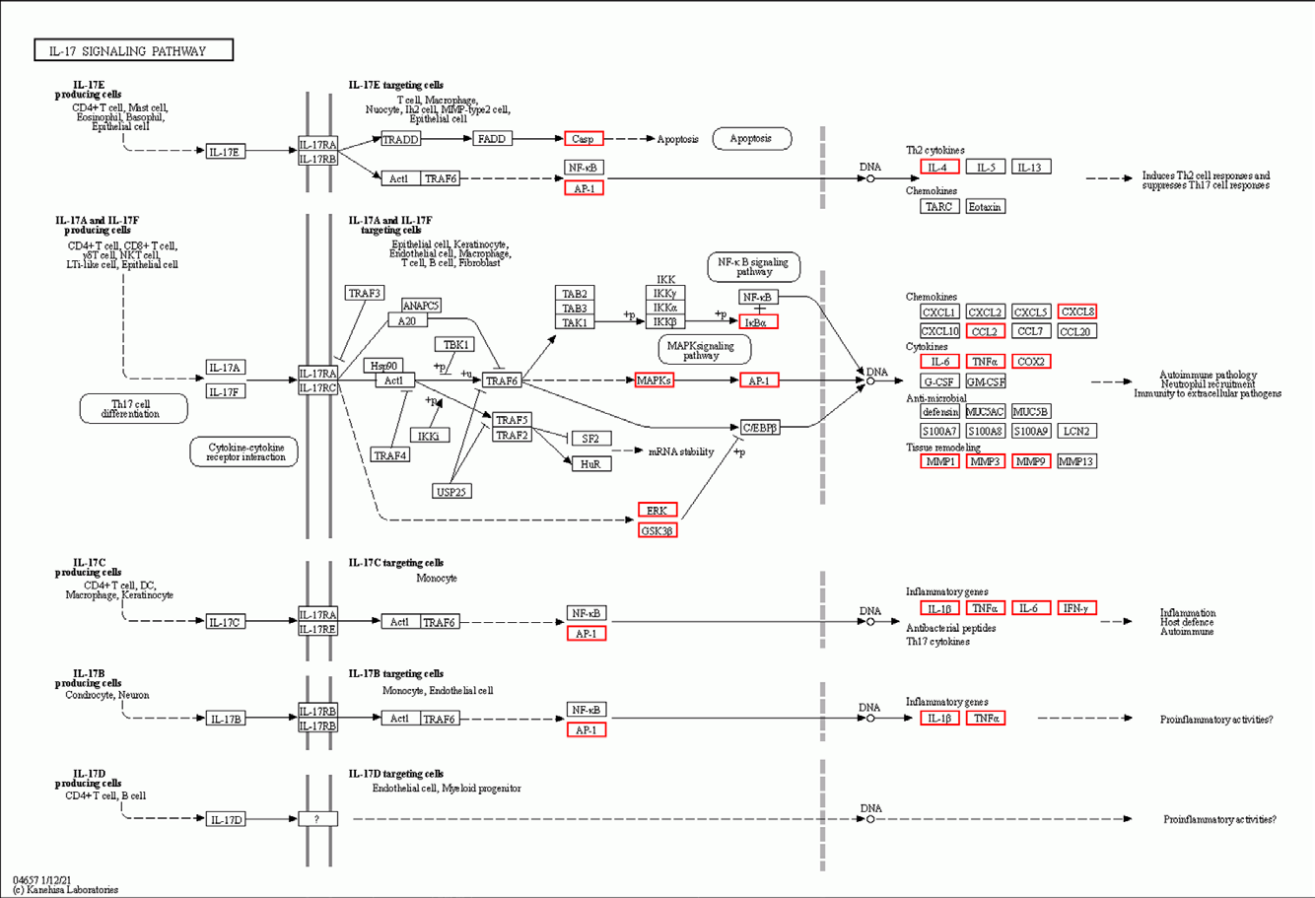
IL-17 pathway (red is the core target).

GO enrichment analysis indicated that GZFZT is involved in multiple biological processes related to LDH, including regulation of apoptotic signaling pathway, response to oxidative stress, reactive oxygen species metabolic process, and muscle cell proliferation. Apoptosis is a programmed cell death process involved in normal development, tissue renewal, and immune system function, and is associated with many physiological and pathological conditions. Currently, 3 common apoptosis-inducing factors—Fas Ligand, TNF-α, and tumor necrosis factor-related apoptosis-inducing ligand (TRAIL)—can trigger apoptosis in intervertebral disc cells through different signaling pathways.^[61–63]^ Studies have shown that Fas can be detected in herniated lumbar disc tissue, with higher expression levels in discs with annulus fibrosus disruption compared to intact discs, and demonstrating that disc cells undergo apoptosis via the FAS and FasL system.^[63]^ The Type II (mitochondrial) pathway, one of the main pathways for Fas-mediated apoptosis, has been shown to occur in disc cell apoptosis.^[64]^ miR-495-3p is a novel miRNA whose overexpression can significantly inhibit TNF-α-induced apoptosis in NP cells.^[62]^ DR4 (death receptor 4) and DR5 (death receptor 5) are TRAIL’s agonistic receptors, and TRAIL binding to either can induce apoptosis.^[65]^ Research has demonstrated that the TRAIL/DR4 pathway is involved in the development of LDH, with DR4 expression positively correlating with disease severity.^[63]^ Additionally, the A20 protein encoded by the tumor necrosis factor α-induced protein 3 (TNFAIP3) gene has been shown to alleviate pain in LDH patients by inhibiting the NF-κB pathway.^[66]^ The KEGG pathway enrichment analysis indicates that GZFZT might be involved in pathways in cancer, lipid and atherosclerosis, AGE-RAGE signaling pathway in diabetic complications, and IL-17 signaling pathway. Notably, the AGE-RAGE signaling pathway, a crucial pathway involved in various biological processes, is found to be dysregulated in LDH as shown in Fig. 10. Carboxymethyl-lysine, one of the main components of advanced glycation end products (AGEs), is derived from glucose and oxidized lipids, and is both stable and irreversible.^[67]^ The receptor for AGEs act as inducers of intracellular pathways, and when carboxymethyl-lysine binds to the receptor for AGEs, NF-κB is released and translocates from the cytoplasm to the nucleus, generating inflammatory signals and oxidative stress responses, inducing the upregulation of MMPs, apoptosis, and other processes. This causes the release of proteolytic enzymes and an imbalance in the metabolism of matrix components, affecting the collagen and elastic connective tissues of the intervertebral disc.^[68,69]^ Recent studies suggest that the excessive accumulation of AGEs in the human body leads to the loss of cell adhesion and increased membrane permeability, resulting in muscle fiber damage.^[70]^ However, it is yet to be determined whether this impacts muscle function and subsequently the intervertebral disc tissue. Studies have found that high levels of IL-17A are associated with lumbar disc degeneration and LDH, and IL-17A is considered a key factor in disc pathology as shown in Fig. 11.^[71]^ IL-17 (also known as IL-17A) is produced by T helper 17 cells, a subset of CD4+ T cells.^[72]^ It has been reported that IL-17A, in conjunction with other cytokines such as TNFα, stimulates the NF-κB pathway, leading to the release of inflammatory cytokines IL-2, IL-6, and TNFα, as well as catabolic enzymes MMP-3, MMP-9, and MMP-13. These factors contribute to the further progression of LDH pathology.^[72–75]^ Compared to healthy controls, LDH patients with NP protrusion combined with AF rupture show increased levels of IL-17A, which correlate with the intensity of sciatic nerve pain.^[76]^ Notably, the frequency of T helper 17 cells and the concentration of IL-17A positively correlate with the visual analog scale scores for low back pain.^[77]^ Although Pathways in cancer and Lipid and atherosclerosis play significant roles in the development of LDH, the current research has limitations, and the mechanisms of LDH remain unclear, necessitating further investigation.

To explore the molecular mechanisms of GZFZT in treating LDH, we employed molecular docking techniques to predict interactions between 6 key target proteins—IL6, MAPK3, STAT3, MAPK1, TNF, and AKT1—and active compounds such as quercetin, kaempferol, 7-methoxy-2-methyl isoflavone, naringenin, formononetin, licochalcone A, isorhamnetin, medicarpin and stigmasterol. Molecular docking results indicate strong binding affinity ranging from −6.9 to −11.0 (kcal/mol) between these proteins and ligands.

Although modern bioinformatics approaches have provided valuable insights into the effectiveness of GZFZT in treating LDH, this study still has certain limitations. Firstly, while network pharmacology and molecular docking techniques have proven to be of great significance, the limitations and delays inherent in network information technology and database data may lead to incomplete or biased research results, thereby affecting the accuracy and reliability of the studies. Therefore, further validation through pharmacokinetic and molecular biology experiments is necessary. Meanwhile, given that network pharmacology research inherently involves highly complex interdisciplinary collaboration, such collaboration inevitably leads to increased research time and costs. Therefore, in future research practices, it is imperative to actively explore and promote opportunities for interdisciplinary cooperation, synergistically accelerating the development of network pharmacology research. Nonetheless, the development of GZFZT holds significant importance and potential, demonstrating substantial therapeutic efficacy and relative safety in the treatment of LDH. Furthermore, the research progress on GZFZT provides a certain decision-making basis for the research directions of scientists and offers inspiration for clinical treatment management.

## 5. Conclusion

In this study, we systematically elucidated the relationship between GZFZT and LDH at the molecular level, indicating the potential of GZFZT as a novel oral drug for the treatment of LDH. Our study identified quercetin, kaempferol, 7-methoxy-2-methylisoflavone, naringenin, β-sitosterol, formononetin, licochalcone A, isorhamnetin, medicarpin, and stigmasterol as the key active components in GZFZT for the treatment of LDH. The mechanism of action involves core targets such as IL6, MAPK3, STAT3, MAPK1, TNF, and AKT1. Notably, naringenin, β-sitosterol, and stigmasterol exhibited prominent targeting effects on MAPK3 in molecular docking studies. Further research is recommended to validate the findings of this study.

## Acknowledgments

The authors acknowledge the Kunming Institute of Botany’s Bioactivity Screening Center for its assistance in evaluating the compounds’ bioactivity.

## Author contributions

**Conceptualization:** Jiafeng Peng.

**Formal analysis:** Jiafeng Peng, Ran Xu.

**Funding acquisition:** Danyang Li.

**Investigation:** Jiafeng Peng, Hongxing Zhang.

**Methodology:** Jiafeng Peng, Li Xia.

**Project administration:** Yingchun Li.

**Resources:** Qianqian Meng.

**Software:** Huaize Wang.

**Supervision:** Minglei Gao, Junchen Zhu.

**Validation:** Xingfu Ma.

**Visualization:** Junchen Zhu.

**Writing – review & editing:** Junchen Zhu.

## Supplementary Material

SUPPLEMENTARY MATERIAL

## References

[1] Zhang AS Xu A Ansari K. Lumbar disc herniation: diagnosis and management. Am J Med. 2023;136:645–51. 37072094 10.1016/j.amjmed.2023.03.024

[2] Wang Z Liu X Gao K Tuo H Zhang X Liu W. Clinical effects and biological mechanisms of exercise on lumbar disc herniation. Front Physiol. 2024;15:1309663. 38292068 10.3389/fphys.2024.1309663 PMC10824856

[3] Chen BL Guo JB Zhang HW. Surgical versus non-operative treatment for lumbar disc herniation: a systematic review and meta-analysis. Clin Rehabil. 2018;32:146–60. 28715939 10.1177/0269215517719952

[4] Chiu CC Chuang TY Chang KH Wu C-H Lin P-W Hsu W-Y. The probability of spontaneous regression of lumbar herniated disc: a systematic review. Clin Rehabil. 2015;29:184–95. 25009200 10.1177/0269215514540919

[5] El Barzouhi A Vleggeert-Lankamp CLAM Nijeholt GJLA. Magnetic resonance imaging in follow-up assessment of sciatica. N Engl J Med. 2013;368:999–1007. 23484826 10.1056/NEJMoa1209250

[6] Luo Y Huang J Xu L Zhao W Hao J Hu Z. Efficacy of Chinese herbal medicine for lumbar disc herniation: a systematic review of randomized controlled trials. J Tradit Chin Med = Chung i tsa chih ying wen pan. 2013;33:721–6. 24660602 10.1016/s0254-6272(14)60003-0

[7] Lee J Lim S. Anti-inflammatory, and anti-arthritic effects by the twigs of cinnamomum cassia on complete freund’s adjuvant-induced arthritis in rats. J Ethnopharmacol. 2021;278:114209. 34015366 10.1016/j.jep.2021.114209

[8] Mai JZ Liu C Huang Z. Oral application of bulleyaconitine a attenuates morphine tolerance in neuropathic rats by inhibiting long-term potentiation at C-fiber synapses and protein kinase C gamma in spinal dorsal horn. Mol Pain. 2020;16:1744806920917242. 32290780 10.1177/1744806920917242 PMC7160774

[9] Yahyazadeh R Baradaran Rahimi V Yahyazadeh A. Promising effects of gingerol against toxins: a review article. Biofactors. 2021;47:885–913. 34418196 10.1002/biof.1779

[10] Lu Y Bao T Mo J Ni J Chen W. Research advances in bioactive components and health benefits of jujube (ziziphus jujuba mill.) fruit. J Zhejiang Univ Sci B. 2021;22:431–49. 34128368 10.1631/jzus.B2000594 PMC8214949

[11] Wang R Chen Y Wang Z. Antidepressant effect of licorice total flavonoids and liquiritin: a review. Heliyon. 2023;9:e22251. 38074876 10.1016/j.heliyon.2023.e22251 PMC10700396

[12] Chen L Yang L Chen RW. Clinical study on the treatment of lumbar disc herniation with Guizhi Fuzi Decoction. Asia-Pacific Tradit Med. 2022;18:87–90. In Chinese.

[13] Wu XC Fang ZL Fang PF. The effect of guizhi fuzi decoction combined with pedicle screw fixation and interbody bone grafting on lumbar disc herniation and pain improvement. Heilongjiang Med J. 2024;37:631–3. In Chinese.

[14] Luan X Zhang LJ Li XQ. Compound-based Chinese medicine formula: from discovery to compatibility mechanism. J Ethnopharmacol. 2020;254:112687. 32105748 10.1016/j.jep.2020.112687

[15] Zhang ND Han T Huang BK. Traditional Chinese medicine formulas for the treatment of osteoporosis: implication for antiosteoporotic drug discovery. J Ethnopharmacol. 2016;189:61–80. 27180315 10.1016/j.jep.2016.05.025

[16] Ru J Li P Wang J. TCMSP: a database of systems pharmacology for drug discovery from herbal medicines. J Cheminf. 2014;6:13. 10.1186/1758-2946-6-13 PMC4001360 24735618

[17] Gu Y Zhu H Wang X Zhang S Tong P Lv S. Exploring the mechanism of buyang huanwu decoction in the treatment of lumbar disc herniation based on network pharmacology and molecular docking. Medicine (Baltimore). 2022;101:e29534. 35960059 10.1097/MD.0000000000029534 PMC9371581

[18] Yang S Jia Y Zhang J Zhai W Xie Y Guo J. A randomized controlled trial: the efficacy and safety of bushen huoxue formula in the management of lower back pain from lumbar disc herniation. Medicine (Baltimore). 2024;103:e37293. 38363892 10.1097/MD.0000000000037293 PMC10869040

[19] Novais EJ Tran VA Johnston SN. Long-term treatment with senolytic drugs dasatinib and quercetin ameliorates age-dependent intervertebral disc degeneration in mice. Nat Commun. 2021;12:5213. 34480023 10.1038/s41467-021-25453-2 PMC8417260

[20] Shao Z Wang B Shi Y. Senolytic agent quercetin ameliorates intervertebral disc degeneration via the Nrf2/NF-κB axis. Osteoarthritis Cartilage. 2021;29:413–22. 33242601 10.1016/j.joca.2020.11.006

[21] Jie J Xu X Li W Wang G. Regulation of apoptosis and inflammatory response in interleukin-1β-induced nucleus pulposus cells by miR-125b-5p via targeting TRIAP1. Biochem Genet. 2021;59:475–90. 33123835 10.1007/s10528-020-10009-8

[22] Imran M Rauf A Shah ZA. Chemo-preventive and therapeutic effect of the dietary flavonoid kaempferol: a comprehensive review. Phytother Res. 2019;33:263–75. 30402931 10.1002/ptr.6227

[23] Calderon-Montano JM Burgos-Moron E Perez-Guerrero C. A review on the dietary flavonoid kaempferol. Mini-Rev Med Chem. 2011;11:298–344. 21428901 10.2174/138955711795305335

[24] Zhu J Tang H Zhang Z. Kaempferol slows intervertebral disc degeneration by modifying LPS-induced osteogenesis/adipogenesis imbalance and inflammation response in BMSCs. Int Immunopharmacol. 2017;43:236–42. 28043032 10.1016/j.intimp.2016.12.020

[25] Mao T Fan J. Myricetin restores autophagy to attenuate lumbar intervertebral disk degeneration via negative regulation of the JAK2/STAT3 pathway. Biochem Genet. 2024. doi: 10.1007/s10528-024-10838-x. 10.1007/s10528-024-10838-x 38842745

[26] Wong SK Chin KY Ima-Nirwana S. The osteoprotective effects of kaempferol: the evidence from in vivo and in vitro studies. Drug Design Develop Ther. 2019;13:3497–514. 10.2147/DDDT.S227738 PMC6789172 31631974

[27] Zhou X Zhang Z Jiang W. Naringenin is a potential anabolic treatment for bone loss by modulating osteogenesis, osteoclastogenesis, and macrophage polarization. Front Pharmacol. 2022;13:872188. 35586056 10.3389/fphar.2022.872188 PMC9108355

[28] Ha H Lee HY Lee JH. Formononetin prevents ovariectomy-induced bone loss in rats. Arch Pharm Res. 2010;33:625–32. 20422373 10.1007/s12272-010-0418-8

[29] Kim SN Kim MH Min YK Kim SH. Licochalcone a inhibits the formation and bone resorptive activity of osteoclasts. Cell Biol Int. 2008;32:1064–72. 18539489 10.1016/j.cellbi.2008.04.017

[30] Tyagi AM Gautam AK Kumar A. Medicarpin inhibits osteoclastogenesis and has nonestrogenic bone conserving effect in ovariectomized mice. Mol Cell Endocrinol. 2010;325:101–9. 20570709 10.1016/j.mce.2010.05.016

[31] Li W Zhao H Zhou S. Does vertebral osteoporosis delay or accelerate lumbar disc degeneration? A systematic review. Osteoporosis Int. 2023;34:1983–2002. 10.1007/s00198-023-06880-x PMC10651704 37578509

[32] Pinho-Ribeiro FA Zarpelon AC Fattori V. Naringenin reduces inflammatory pain in mice. Neuropharmacology. 2016;105:508–19. 26907804 10.1016/j.neuropharm.2016.02.019

[33] Hu CY Zhao YT. Analgesic effects of naringenin in rats with spinal nerve ligation-induced neuropathic pain. Biomed Reports. 2014;2:569–73. 10.3892/br.2014.267 PMC4051469 24944810

[34] Oliveira MA Heimfarth L Passos FRS. Naringenin complexed with hydroxypropyl-β-cyclodextrin improves the sciatic nerve regeneration through inhibition of p75NTR and JNK pathway. Life Sci. 2020;241:117102. 31790691 10.1016/j.lfs.2019.117102

[35] Lim JH Kim SE Kim HJ Song GG Jung JH. Intra-articular injection of stigmasterol-loaded nanoparticles reduce pain and inhibit the inflammation and joint destruction in osteoarthritis rat model: a pilot study. Drug Delivery Translat Res. 2024;14:1969–81. 10.1007/s13346-023-01501-w 38200400

[36] Li K Li S Zhang H Lei D Lo WLA Ding M. Computational analysis of the immune infiltration pattern and candidate diagnostic biomarkers in lumbar disc herniation. Front Mol Neurosci. 2022;15:846554. 35531067 10.3389/fnmol.2022.846554 PMC9069112

[37] Piccio L Vermi W Boles KS. Adhesion of human T cells to antigen-presenting cells through SIRPbeta2-CD47 interaction costimulates T-cell proliferation. Blood. 2005;105:2421–7. 15383453 10.1182/blood-2004-07-2823

[38] Zani I Stephen S Mughal N. Scavenger receptor structure and function in health and disease. Cells. 2015;4:178–201. 26010753 10.3390/cells4020178 PMC4493455

[39] Riuzzi F Sorci G Arcuri C. Cellular and molecular mechanisms of sarcopenia: the S100B perspective. J Cachexia Sarcopenia Muscle. 2018;9:1255–68. 30499235 10.1002/jcsm.12363 PMC6351675

[40] Li D Li K Chen G. S100B suppresses the differentiation of C3H/10T1/2 murine embryonic mesenchymal cells into osteoblasts. Mol Med Rep. 2016;14:3878–86. 27601207 10.3892/mmr.2016.5697

[41] Ren X Geng M Xu K. Quantitative proteomic analysis of synovial tissue reveals that upregulated OLFM4 aggravates inflammation in rheumatoid arthritis. J Proteome Res. 2021;20:4746–57. 34496567 10.1021/acs.jproteome.1c00399

[42] Zhao Y Qiu C Wang W. Cortistatin protects against intervertebral disc degeneration through targeting mitochondrial ROS-dependent NLRP3 inflammasome activation. Theranostics. 2020;10:7015–33. 32550919 10.7150/thno.45359 PMC7295059

[43] Risbud MV Shapiro IM. Role of cytokines in intervertebral disc degeneration: pain and disc content. Nat Rev Rheumatol. 2014;10:44–56. 24166242 10.1038/nrrheum.2013.160 PMC4151534

[44] Ye W Zhou J Markova DZ. Xylosyltransferase-1 expression is refractory to inhibition by the inflammatory cytokines tumor necrosis factor α and IL-1β in nucleus pulposus cells. Am J Pathol. 2015;185:485–95. 25476526 10.1016/j.ajpath.2014.09.021 PMC4305180

[45] Huang Y Wang L Luo B. Associations of lumber disc degeneration with paraspinal muscles myosteatosis in discogenic low back pain. Front Endocrinol. 2022;13:891088. 10.3389/fendo.2022.891088 PMC9136003 35634490

[46] Scheller J Chalaris A Schmidt-Arras D Rose-John S. The pro- and anti-inflammatory properties of the cytokine interleukin-6. Biochim Biophys Acta. 2011;1813:878–88. 21296109 10.1016/j.bbamcr.2011.01.034

[47] Rand N Reichert F Floman Y Rotshenker S. Murine nucleus pulposus-derived cells secrete interleukins-1-beta, -6, and -10 and granulocyte-macrophage colony-stimulating factor in cell culture. Spine (Phila Pa 1976). 1997;22:2598–601; discussion 2602. 9399443 10.1097/00007632-199711150-00002

[48] Zhao QX Wang YH Wang SC Xue S Cao Z-X Sun T. Protectin DX Attenuates lumbar radicular pain of non-compressive disc herniation by autophagy flux stimulation via adenosine monophosphate-activated protein kinase signaling. Front Physiol. 2022;12:784653. 35069245 10.3389/fphys.2021.784653 PMC8770935

[49] Murata Y Rydevik B Nannmark U. Local application of interleukin-6 to the dorsal root ganglion induces tumor necrosis factor-alpha in the dorsal root ganglion and results in apoptosis of the dorsal root ganglion cells:. Spine. 2011;36:926–32. 21192292 10.1097/BRS.0b013e3181e7f4a9

[50] Murata Y Nannmark U Rydevik B. The role of tumor necrosis factor-alpha in apoptosis of dorsal root ganglion cells induced by herniated nucleus pulposus in rats. Spine (Phila Pa 1976). 2008;33:155–62. 18197099 10.1097/BRS.0b013e3181605518

[51] Osuka K Usuda N Aoyama M. Expression of the JAK/STAT3/SOCS3 signaling pathway in herniated lumbar discs. Neurosci Lett. 2014;569:55–8. 24686183 10.1016/j.neulet.2014.03.045

[52] Cao S Han X Ding C. Molecular cloning of the duck mitogen-activated protein kinase 1 (MAPK1) gene and the development of a quantitative real-time PCR assay to detect its expression. Poult Sci. 2014;93:2158–67. 24974389 10.3382/ps.2013-03796

[53] Wuertz K Vo N Kletsas D Boos N. Inflammatory and catabolic signalling in intervertebral discs: the roles of NF-κB and MAP kinases. Eur Cell Mater. 2012;23:103–19; discussion 119. 22354461 10.22203/ecm.v023a08

[54] Zhu J Zhang X Gao W Hu H Wang X Hao D. lncRNA/circRNA-miRNA-mRNA ceRNA network in lumbar intervertebral disc degeneration. Mol Med Rep. 2019;20:3160–74. 31432173 10.3892/mmr.2019.10569 PMC6755180

[55] Zhou M He S Liu W. EZH2 upregulates the expression of MAPK1 to promote intervertebral disc degeneration via suppression of miR‐129‐5p. J Gene Med. 2022;24:e3395. 34668273 10.1002/jgm.3395

[56] Noguchi M Ropars V Roumestand C Suizu F. Proto‐oncogene TCL1: more than just a coactivator for akt. FASEB J. 2007;21:2273–84. 17360849 10.1096/fj.06-7684com

[57] Manning BD Toker A. AKT/PKB signaling: navigating the network. Cell. 2017;169:381–405. 28431241 10.1016/j.cell.2017.04.001 PMC5546324

[58] Chen J Somanath PR Razorenova O. Akt1 regulates pathological angiogenesis, vascular maturation and permeability in vivo. Nat Med. 2008;11:1188–96. 10.1038/nm1307 PMC2277080 16227992

[59] Ozaki CK. Akt1/protein kinase bα is critical for ischemic and VEGF-mediated angiogenesis. Yearbook Vasc Surg. 2007;2007:37–8.

[60] Pasku D Soufla G Katonis P Tsarouhas A Vakis A Spandidos DA. Akt/PKB isoforms expression in the human lumbar herniated disc: correlation with clinical and MRI findings. Eur Spine J. 2011;20:1676–83. 21590431 10.1007/s00586-011-1841-3 PMC3175883

[61] Park JB Chang H Kim KW. Expression of fas ligand and apoptosis of disc cells in herniated lumbar disc tissue:. Spine. 2001;26:618–21. 11246372 10.1097/00007632-200103150-00011

[62] Lin X Lin Q. MiRNA-495-3p attenuates TNF-α induced apoptosis and inflammation in human nucleus pulposus cells by targeting IL5RA. Inflammation. 2020;43:1797–805. 32445070 10.1007/s10753-020-01254-5

[63] Zhang L Niu T Yang SY Lu Z Chen B. The occurrence and regional distribution of DR4 on herniated disc cells: a potential apoptosis pathway in lumbar intervertebral disc. Spine. 2008;33:422–7. 18277875 10.1097/BRS.0b013e318163e036

[64] Park JB Lee JK Park SJ Kim KW Riew KD. Mitochondrial involvement in fas-mediated apoptosis of human lumbar disc cells. J Bone Joint Surg Am. 2005;87:1338–42. 15930545 10.2106/JBJS.D.02527

[65] Chaudhary PM Eby M Jasmin A Bookwalter A Murray J Hood L. Death receptor 5, a new member of the TNFR family, and DR4 induce FADD-dependent apoptosis and activate the NF-kappaB pathway. Immunity. 1997;7:821–30. 9430227 10.1016/s1074-7613(00)80400-8

[66] Xie Z Chen J Xiao Z Li Y Yuan T Li Y. TNFAIP3 alleviates pain in lumbar disc herniation rats by inhibiting the NF-κB pathway. Ann Translat Med. 2022;10:80. 10.21037/atm-21-6499 PMC8848453 35282077

[67] Kang J Jeong YJ Ha SK Lee HH Lee K-W. Glyoxal-derived advanced glycation end-products, nε-carboxymethyl-lysine, and glyoxal-derived lysine dimer induce apoptosis-related gene expression in hepatocytes. Mol Biol Rep. 2023;50:2511–20. 36609749 10.1007/s11033-022-08130-5

[68] Haslbeck KM Friess U Schleicher ED. The RAGE pathway in inflammatory myopathies and limb girdle muscular dystrophy. Acta Neuropathol. 2005;110:247–54. 15986224 10.1007/s00401-005-1043-3

[69] Eiras S. Nε-carboxymethyl-lysine and inflammatory cytokines, markers and mediators of coronary artery disease progression in diabetes. World J Diabetes. 2024;15:575–8. 38680703 10.4239/wjd.v15.i4.575 PMC11045414

[70] Egawa T Ogawa T Yokokawa T. Glycative stress inhibits hypertrophy and impairs cell membrane integrity in overloaded mouse skeletal muscle. J Cachexia Sarcopenia Muscle. 2024;15:883–96. 38575520 10.1002/jcsm.13444 PMC11154761

[71] Suyama K Sakai D Hirayama N. Effects of interleukin‐17A in nucleus pulposus cells and its small‐molecule inhibitors for intervertebral disc disease. J Cell Mol Med. 2018;22:5539–51. 30207057 10.1111/jcmm.13828 PMC6201370

[72] Gaffen SL. Recent advances in the IL-17 cytokine family. Curr Opin Immunol. 2011;23:613–9. 21852080 10.1016/j.coi.2011.07.006 PMC3190066

[73] Hartupee J Liu C Novotny M Li X Hamilton T. IL-17 enhances chemokine gene expression through mRNA stabilization. J Immunol (Baltimore, Md. : 1950). 2007;179:4135–41. 10.4049/jimmunol.179.6.4135 17785852

[74] Onishi RM Gaffen SL. Interleukin‐17 and its target genes: mechanisms of interleukin‐17 function in disease. Immunology. 2010;129:311–21. 20409152 10.1111/j.1365-2567.2009.03240.x PMC2826676

[75] Zhang G Liu M Chen H. NF‐κB signalling pathways in nucleus pulposus cell function and intervertebral disc degeneration. Cell Prolif. 2021;54:e13057. 34028920 10.1111/cpr.13057 PMC8249791

[76] Cheng L Fan W Liu B Wang X Nie L. Th17 lymphocyte levels are higher in patients with ruptured than non-ruptured lumbar discs, and are correlated with pain intensity. Injury. 2013;44:1805–10. 23680281 10.1016/j.injury.2013.04.010

[77] Zhang W Nie L Guo YJ. Th17 cell frequency and IL-17 concentration correlate with pre- and postoperative pain sensation in patients with intervertebral disk degeneration. Orthopedics. 2014;37:e685–91. 10.3928/01477447-20140626-62 24992069

